# LPX-TI641, a Tim3/4 Agonist, Induces Long-Term Immune Tolerance in Multiple Sclerosis Models

**DOI:** 10.3390/pharmaceutics17111402

**Published:** 2025-10-30

**Authors:** Anas M. Fathallah, Abdulraouf Ramadan, Basel Karzoun, Hannah Leahy, Nimita Dave, Raed Khashan, Saleh Allababidi, Shiv Saidha, Sarah Madani

**Affiliations:** 1 LAPIX Therapeutics Inc., Cambridge, MA 021141, USA; 2 Artelligence Therapeutics LLC, Philadelphia, PA 19114, USA; raed.khashan@gmail.com; 3 Division of Neuroimmunology and Neurological Infections, Department of Neurology, Johns Hopkins University, Baltimore, MD 21218, USA; 4 Department of Neurology, Atrius Health, Boston, MA 02215, USA; 5 Department of Neurology, Beth Israel Deaconess Medical Center, Boston, MA 02215, USA

**Keywords:** Regulatory T-cells (Tregs), immune tolerance, multiple sclerosis, immune regulation, oral therapy, drug discovery

## Abstract

**Background**: Current disease-modifying therapies (DMTs) for multiple sclerosis (MS) attenuate pathogenic immune responses but are limited by safety and tolerability concerns. Antigen-specific tolerance approaches provide targeted immunomodulation yet remain constrained by their dependence on known autoantigens. LPX-TI641, an orally bioavailable, clinical-stage small-molecule agonist of Tim-3/4, represents an antigen-independent strategy to restore immune tolerance by expanding regulatory T cells (Tregs). **Methods**: LPX-TI641 was evaluated in vitro for its ability to induce Treg populations in murine splenocytes. Therapeutic efficacy was assessed in vivo using MOG_35–55_- and PLP_139–151_-induced experimental autoimmune encephalomyelitis (EAE) mouse models. Ex vivo, peripheral blood mononuclear cells (PBMCs) from people with MS (PwMS) were analyzed for Treg phenotype and function in response to LPX-TI641. **Results**: LPX-TI641 induced dose-dependent expansion of CD4^+^Foxp3^+^ and CD4^+^Foxp3^+^Tim-3^+^ Tregs in vitro. In EAE models, treatment significantly reduced disease severity, prevented relapses, and maintained clinical benefit after discontinuation. In PBMCs from patients with MS, LPX-TI641 restored diminished Tim-3^+^ Treg populations and reversed Treg dysfunction in recall assays. Efficacy in animal models was comparable to or exceeded that of high-efficacy DMTs, including natalizumab. **Conclusions**: LPX-TI641 promotes antigen-independent immune tolerance through Tim receptor agonism and Treg expansion. These findings support its potential as a novel therapeutic candidate for MS, addressing the limitations of current DMTs.

## 1. Introduction

Multiple sclerosis (MS) is a chronic autoimmune disease of the central nervous system, driven by a breakdown in immune tolerance that leads to inflammation, demyelination, and neurodegeneration. This dysregulation is marked by an expansion of autoreactive Th1 and Th17 cells and a concurrent reduction in regulatory T and B cell subsets [1,2,3]. While current disease-modifying therapies (DMTs) effectively suppress inflammation, they target downstream immune responses and often cause broad immunosuppression, increasing the risk of infections and other serious adverse events [4,5].

The clinical management of MS typically follows one of two paradigms: an escalation approach starting with lower-risk therapies and progressing to more potent treatments as needed, or an induction/maintenance approach using high-efficacy therapies early to establish disease control before transitioning to a maintenance regimen [6]. Approved DMTs span multiple mechanisms, including immunomodulators (e.g., glatiramer acetate, dimethyl fumarate), immune cell-depleting agents (e.g., alemtuzumab, ocrelizumab), anti-proliferative agents (e.g., teriflunomide), and migration inhibitors (e.g., natalizumab, fingolimod) [7]. Despite their diversity, these therapies share a limitation: they act downstream of the root immunological defect, loss of tolerance and their use is often limited by tolerability and safety concerns, including risks of infections and other serious adverse events often leading to discontinuation and switching between different therapeutic classes [4,5].

Given these limitations, there is an unmet medical need in the clinical management of MS and other autoimmune diseases to address the root cause of autoimmunity, the loss of immune tolerance, without inducing broad immunosuppression. One promising approach is the induction of immune tolerance.

Antigen-specific immune tolerance strategies have shown promise in both preclinical and clinical settings [8,9]. However, its practical application has been hampered by the requirement to present disease-relevant antigens, which in many autoimmune diseases are often multiple and poorly defined, thus complicating the implementation of antigen-specific therapies.

The Tim family of receptors, including Tim-3 and Tim-4, plays a central role in maintaining immune homeostasis by regulating inflammation and promoting tolerance across autoimmune, allergic, and transplant settings. Tim-3, in particular, engages ligands such as galectin-9 and phosphatidylserine to suppress pathogenic Th1/Th17 responses and enhance regulatory T cell (Treg) function. Blockade of Tim-3 exacerbates disease in models like EAE, underscoring its role as a negative regulator of immune activation. Both Tim-3 and Tim-4 contribute to the expansion of regulatory T and B cells, supporting the use of Tim receptor agonism as a non-antigen-specific strategy for inducing immune tolerance in autoimmune diseases such as multiple sclerosis (MS) [10,11,12,13].

We have previously reported on the use of LPX3, a T cell immunoglobulin and mucin domain (Tim) family receptors agonist, to induce antigen-specific tolerance toward AAV capsid proteins in the context of gene therapy [14] and to promote antigen-agnostic immune tolerance in MOG_35–55_ induced EAE mouse models [15].

Here, we present LPX-TI641, a novel, orally bioavailable, clinical-stage small-molecule agonist of Tim-3 and Tim-4 that is more potent than LPX3. We evaluated LPX-TI641 in vitro and in vivo, assessing its ability to expand CD4^+^Foxp3^+^ regulatory T cells (Tregs), restore Tim-3^+^ Tregs in peripheral blood mononuclear cells (PBMCs) from people with MS (PwMS), and improve suppressive Treg function. In EAE models, LPX-TI641 significantly reduced clinical scores and prevented relapses. These findings support the therapeutic potential of LPX-TI641 as a non-antigen-specific immune tolerance strategy for MS and related autoimmune conditions.

## 2. Materials and Methods

### 2.1. Docking Simulations

#### 2.1.1. Preparation for Docking

The co-crystal structure of the Tim protein bound to its endogenous ligand, dicaproyl-phosphatidylserine (PS) was used for docking simulations. The ligand (PS) was removed from the binding site to allow docking of the proposed molecules. All water molecules were removed except for one, which was essential for forming a hydrogen bond bridge between PS and the protein. Charges for all atoms in the protein were calculated at a physiological pH of 7.4, matching the conditions used in the in vitro experiments. Similarly, charges for all docked molecules were also computed at the same physiological pH. Docking was performed using rigid binding, and the top 20 binding poses for each molecule were identified for further analysis.

#### 2.1.2. Validation of the Docking Method

To validate the docking approach, the endogenous ligand (PS) was re-docked into the protein’s binding site after its removal. The root mean- square deviation (RMSD) of the pose that matched the native configuration was calculated and ranked to demonstrate the reliability of the docking procedure.

#### 2.1.3. Docking of Experimentally Tested and Proposed Structures

After validating the docking method, several compounds (PS, LPX-TI641 and LPX3) were docked into the Tim receptor’s binding site using rigid docking methods. The binding affinity of the best-scoring pose (ΔG of binding in kcal/mol) was recorded. Based on the ΔG values, theoretical dissociation constants (Kd) were calculated at 298 K (250C).

### 2.2. Institutional Animal Care and Use Committee

All animal procedures were approved by the Institutional Animal Care and Use Committee (IACUC) at CRADL Charles River, Boston, MA (protocol number EB17-029-303). Mice were housed under a 12-h light/dark cycle with ad libitum access to food and water.

### 2.3. Animals

Female C57BL/6, C57BL/6 Foxp3^GFP^, SJL, or CD1 mice (7–8 weeks old) were purchased from Charles River Laboratories (Wilmington, MA, USA) and maintained under specific pathogen-free (SPF) conditions at the LAPIX Therapeutics animal facility, managed by CRADL Charles River.

### 2.4. Phosphatidylserine (PS) Liposome Preparation

PS liposomes were prepared using the thin-film hydration method. PS was dissolved in chloroform and mixed with DMPC (Avanti Polar Lipids, Alabaster, AL, USA) at a 30:70 molar ratio in a round-bottom flask. The solvent was evaporated under vacuum using a rotary evaporator to form a lipid film. The film was hydrated with PBS, followed by extrusion through a 0.1 µm filter to generate uniform liposomes.

### 2.5. In Vitro Pharmacology

#### 2.5.1. Dose–Response Relationship

Splenocytes from C57BL/6 mice were cultured in complete DMEM supplemented with 10% FBS, 1% penicillin–streptomycin, 1% L-glutamine, and 0.1% 2-mercaptoethanol (ThermoFisher Scientific, Waltham, MA, USA). Cells were seeded in round-bottom 96-well plates (1 × 10^6^ cells/mL). Cells were treated with increasing concentrations of LPX-TI641 (1–600 nM) or PS liposomes (1–600 nM). Cells were cultured for five days at 37 °C, 5% CO_2_, and analyzed for immune responses.

#### 2.5.2. Tim-3 and Tim-4 Blocking Studies

Splenocytes from C57BL/6 mice were pre-incubated with 20 µg/mL α-Tim-3 (clone 8B.2C12, ThermoFisher Scientific, Waltham, MA, USA) or 20 µg/mL α-Tim-4 (clone RMT4-54, ThermoFisher Scientific) before treatment with increasing concentration of LPX-TI641 as above. Cells were cultured for five days at 37 °C, 5% CO_2_, and analyzed for immune responses.

#### 2.5.3. T-Reg Suppressive Assay

T-regs and CD8+ T cells were isolated using magnetic bead-based kits from StemCell Technologies (Vancouver, BC, USA). T-regs were obtained from splenic C57Bl/6 CD45.1 mice treated with either a single 10 µg subcutaneous (SC) injection of LPX-TI641, from mice administered 500 ng/day of IL-2 intraperitoneally (IP) for 5 consecutive days, or mice administered PBS as control. CD8+ T cells were isolated from spleens of C57Bl/6 CD45.2 mice and labeled with CellTrace Violet, following the manufacturer’s protocol. For co-culture experiments, T-regs and CD8+ T cells were combined at ratios of 1:1, 1:2, 1:4, 1:8, 1:16 and 1:32 of T-reg: CD8 in the presence of 1 µg/mL α-CD3 and 10 µg/mL α-CD28 antibodies. Co-cultures were maintained for 3 days in complete DMEM supplemented with 10% FBS, 2 mM L-glutamine, and 100 U/mL penicillin–streptomycin. At the end of the culture period, cells were washed and stained with CD45.2, CD8, and a viability dye. CD8+ T cell proliferation was evaluated by measuring the dilution of CellTrace Violet using flow cytometry.

### 2.6. Pharmacokinetics and In Vivo Dose Response Studies

#### 2.6.1. Pharmacokinetics Studies of LPX-TI641

CD1 mice were divided into two groups and dosed with 0.15 mg/kg of LPX-TI641 intravenously (IV, *n* = 27) or 0.5 mg/kg orally (PO, *n* = 24). PK samples were collected at predetermined time points: 0.085, 0.25, 0.5, 1, 2, 4, 6, 8, and 10 h post-dose for the IV group, and 0.25, 0.5, 1, 2, 4, 6, 8, and 10 h post-dose for the PO group (*n* = 3 per time point).

Plasma concentrations were measured using a qualified LC-MS/MS method. Non-compartmental analysis (NCA) was performed using Phoenix (version 8.3.4.295) to obtain AUC_last_ and AUC_∞_. The dosing option was set to “Extravascular” for PO and “IV Bolus” for IV. Linear trapezoidal interpolation and uniform weighting were applied. Bioavailability was calculated as the dose-corrected ratio of AUCs for PO versus IV.

#### 2.6.2. Dose–Response Data for LPX-TI641 in C57BL/6 Mice

Female C57BL/6 mice (*n* = 3 per group) were housed under standard conditions and received a single SC dose of 0.01, 0.1, 1, 10, 30 or 100 µg of LPX-TI641 per mouse. Five days post-dose, animals were euthanized, and spleens were collected for T-cell phenotyping. Spleens were prepared into a single-cell suspension and were stained for viability, TCR, CD4, and Foxp3. Cells were gated for TCR^+^CD4^+^ and percent CD4^+^Foxp3^+^ T-cells were evaluated using flow cytometry.

Dose–response data were analyzed using four-parameter logistic regression in R (version 4.5.0) with the *drc* package (version 3.0-1) to estimate ED_50_ and ED_90_ values.

### 2.7. Experimental Autoimmune Encephalomyelitis (EAE)

#### 2.7.1. Dose-Ranging Studies in High Dose MOG_35–55_ EAE Model

EAE was induced in mice by subcutaneous immunization with 100 µg of MOG_35–55_ peptide (R&D Systems, Minneapolis, MN, USA, cat# 25681) emulsified in Complete Freund’s Adjuvant (CFA; ThermoScientific, cat# 77140). Pertussis toxin (200 ng/mouse; List Biological Labs, Campbell, CA, USA, cat# NC0830484) was administered intraperitoneally on day 0 and day 2.

Clinical scoring began on day 7 and continued daily until study completion using the following scoring system: 0: No clinical signs, 1: Tail paralysis, 2: Hind limb weakness or loss of righting reflex. Alternatively, the mouse appears normal (score 0) but exhibits obvious head tilting during walking, indicating poor balance, 2.5: One hind limb paralyzed, 3: Two hind limbs paralyzed, 3.5: Two hind limbs paralyzed and one front limb paralyzed, 4: Quadriplegia, 5: Found dead in cage.

**Figure 1:**
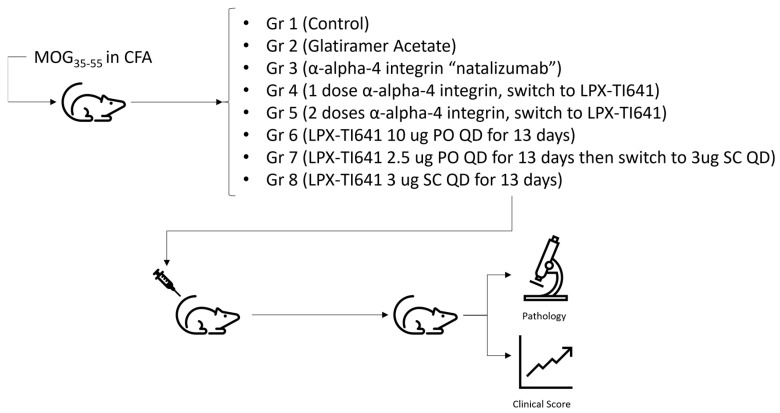
Experimental design and treatment paradigm for EAE induction and LPX-TI641 intervention.

Once mice reached a clinical score of 1, they were randomized into treatment groups (*n* = 8/group) using a random number generator. Treatment groups included vehicle control, glatiramer acetate (100 µg SC daily; MedChem Express, Monmouth Junction, NJ, USA, cat# HY-109520), natalizumab (1.5 mg IV at disease onset and again on day 3; MedChem Express, cat# HY-108831), and several LPX-TI641 regimens as detailed in (Figure 1).

Selected mice were sacrificed and perfused with PBS at the end of study. Spinal cords were processed for histological analysis using Luxol Fast Blue staining to assess myelin integrity and neuronal structure as described in [16].

#### 2.7.2. Dose–Response Studies in PLP139-151 EAE Model

Mice were immunized subcutaneously with 100 µg of PLP_139–151_ peptide (R&D Systems, cat# 2567/1) emulsified in Complete Freund’s Adjuvant (CFA; ThermoScientific, cat# 77140). Clinical signs developed between days 9 and 12. Daily monitoring and clinical scoring began on day 7 and continued throughout the study. On day 15, when the average clinical score reached ≤ 2.5, mice were randomized into five treatment groups: control (*n* = 9), natalizumab (*n* = 14; 0.1 mg IV on days 15, 18, and 21), natalizumab switch (*n* = 9; 0.1 mg IV on day 15 followed by 3 µg LPX-TI641 subcutaneously once daily from days 18 to 26), LPX-TI641 (*n* = 15; 3 µg SC daily from days 15 to 26), and LPX-TI641 (*n* = 10; 1 µg SC daily from days 15 to 26).

On day 21, one mouse per group (selected based on average clinical score) was sacrificed for brain and spleen collection. Brain tissues were processed into single-cell suspensions for flow cytometry analysis.

#### 2.7.3. Efficacy in an Escalation Treatment Paradigm vs. Natalizumab and DMF

**Figure 2:**
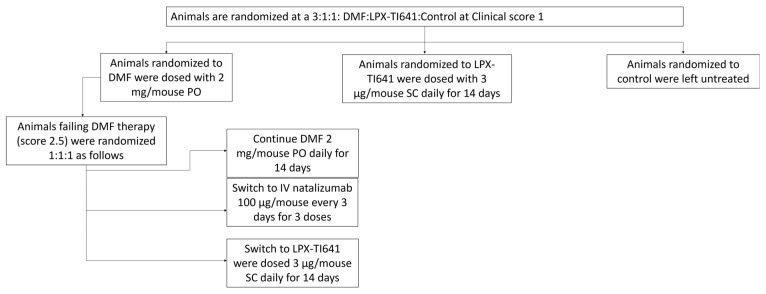
Study design and treatment timeline for the MOG_35–55_ induced EAE model in a therapeutic escalation treatment paradigm.

EAE was induced in mice as previously described. Daily clinical scoring began on day 7 and continued throughout the study, with symptom onset occurring between days 9 and 14. Once mice reached a clinical score of 1, they were randomized at a 3:1:1 ratio into: DMF (2 mg PO QD, Sigma, cat# 64625-100G-F), LPX-TI641 (sustained-use treatment groups, 3 µg SC QD for 14 days), or control as shown in Figure 2.

Within the DMF group, animals that failed treatment—defined as receiving a clinical score of 2.5—were further randomized at a 1:1:1 ratio into an escalation cohort. These mice either continued DMF (2 mg/mouse PO QD for 14 days), switched to natalizumab (100 µg/mouse IV every three days for three doses; MedChem Express, cat# HY-108831), or switched to LPX-TI641 (3 µg/mouse SC QD for 14 days) (Figure 2).

Survival analysis included animals from all cohorts. EAE-related death (defined as a clinical score of 5) was recorded as an event (“1”), while deaths due to ulceration, sacrifice for ex vivo analysis, or survival to day 27 were censored (“0”). Survival data were analyzed using GraphPad Prism 9.

#### 2.7.4. Efficacy in Induction/Maintenance Paradigm vs. Natalizumab and DMF

**Figure 3:**
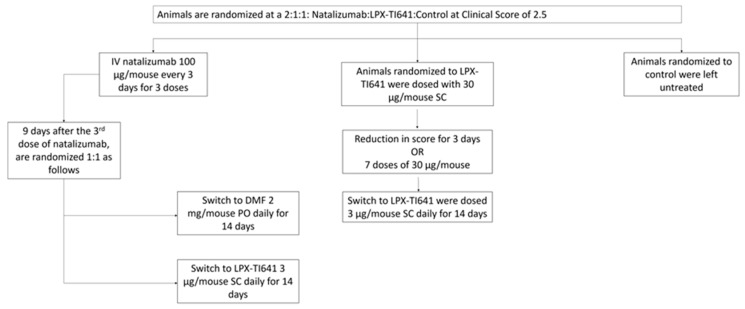
Study design and treatment timeline for the MOG_35–55_ induced EAE model using an induction/maintenance treatment paradigm.

EAE was induced in mice as previously described in Section 2.7.1. Daily clinical scoring began on day 7 and continued for the duration of the study. Symptoms typically developed between days 9 and 14. Once mice reached a clinical score of 2.5, they were randomized in a 2:1:1 ratio into natalizumab, LPX-TI641, or control groups, collectively referred to as the induction cohort (Figure 3).

Mice in the LPX-TI641 group received 30 µg per mouse subcutaneously once daily for a maximum of 7 days or until disease control was achieved, defined as seven consecutive days without a change in clinical score. Upon achieving disease control, animals transitioned to a maintenance dose of 3 µg LPX-TI641 SC daily for 14 days. Natalizumab-treated animals received 100 µg IV every three days for a total of three doses. Once disease control was reached, they were randomized 1:1 to receive either dimethyl fumarate (2 mg/mouse orally once daily; Sigma Aldrich, cat# 242926-100G) or LPX-TI641 (3 µg/mouse SC once daily). All animals were monitored for an additional 14 days following their final dose to assess disease progression and survival outcomes (Figure 3).

### 2.8. Single-Cell Preparation from Mouse Spleen and Brain

#### 2.8.1. Spleen

Spleens were harvested under sterile conditions, crushed over a 70 µm cell strainer, and resuspended in DMEM with 10% FBS. Red blood cells were lysed with lysis buffer (Thermo Fisher Scientific) for 5 min at room temperature, and the remaining cells were washed and resuspended in complete DMEM.

#### 2.8.2. Brain

Mice were euthanized following intracardiac perfusion with cold PBS under isoflurane anesthesia. Brains were rapidly harvested, minced, and digested with 30 U/mL collagenase B (Roche) and 1 mg/mL DNase I (Roche) at 37 °C for 30 min. The tissue was filtered through a 70 µm cell strainer, centrifuged, and processed with Percoll gradients (30%/70%) to isolate mononuclear cells for flow cytometry as described in [17].

### 2.9. Immunostaining and Flow Cytometry

Cells prepared in vitro or isolated ex vivo from spleen or brain were washed and pre-incubated with anti-mouse CD16/CD32 (clone 93, ThermoFisher Scientific) for 10–20 min at 4 °C to block Fc receptors. Surface staining was performed using fluorochrome-conjugated antibodies against CD3 and CD4 (clone GK1.5) for 30 min at 4 °C. After washing with PBS, cells were incubated with Fixable Viability Dye (ThermoFisher Scientific) to assess viability. For intracellular staining, cells were fixed and permeabilized using the Transcription Factor Buffer Set (ThermoFisher Scientific) according to the manufacturer’s instructions, followed by staining for Foxp3 (clone FJK-16s). Cells were then washed, fixed, and analyzed on an Attune NxT Flow Cytometer (ThermoFisher Scientific). Data was processed using FlowJo v10 (TreeStar, Ashland, OR, USA).

### 2.10. PBMCs from Patient with MS (PwMS) Cultured with LPX-TI641

#### 2.10.1. Clinical Samples

A hundred mL of whole blood from six PwMS was acquired via Sanguine Biosciences (Woburn, MA, USA) under their protocols and IRB approvals. PBMCs were collected from whole blood were isolated and drawn by Ficoll (GE Healthcare, Chicago, IL, USA) density gradient centrifugation before being used in subsequent in vitro studies.

#### 2.10.2. PwMS-Derived PBMCs Culture with LPX-TI641

Isolated PBMCs were cultured for 5 days in the presence of increasing concentrations of LPX-TI641, with or without 100 µg/mL of myelin basic protein peptide MBP_87–99_. At the end of the incubation period, cells were harvested, washed, and stained for flow cytometric analysis.

### 2.11. Statistics

Unless otherwise specified, data are shown as either means ± standard deviation (SD) from a single representative experiment with triplicates. All statistical analyses were conducted using the GraphPad Prism program version 10. Comparisons between treatment and control groups were made using unpaired t tests. For 3 groups or more, 1- or 2-way ANOVA with multiple comparison to the control was used, correction for multiple comparison (Tukey) was used as appropriate. A *p*-value less than 0.05, 0.01, 0.001, or 0.0001 is shown as *, **, ***, or ****, respectively.

## 3. Results

### 3.1. Improved Tim Binding of LPX-TI641 vs. LPX3 and PS

#### 3.1.1. Validation of Docking Model

Redocking the natural ligand resulted in poses that matched the native binding configuration with a root mean square deviation (RMSD) of 2.213 Å and ranked among the top 11 poses, demonstrating the reliability of the docking procedure. The computationally calculated binding affinity of PS to its protein was found to be −6.0 kcal/mol.

#### 3.1.2. Docking New Drug Candidates

In silico binding affinity values of rigid docking for the top 10 binding poses showed that LPX-TI641 binding was between 1.2 And 2.2 and 1.7 to 3.0 kcal/mol lower than Tim’s natural lipidic ligand PS and LPX3, respectively (Table 1). This represents a Kd value for LPX-TI641 that is 2 and 3 orders of magnitude lower than PS and LPX3 respectively (i.e., better binding).

### 3.2. In Vitro Pharmacology

#### 3.2.1. LPX-TI641 Is a More Potent Tim Activator than Phosphatidylserine

**Figure 4:**
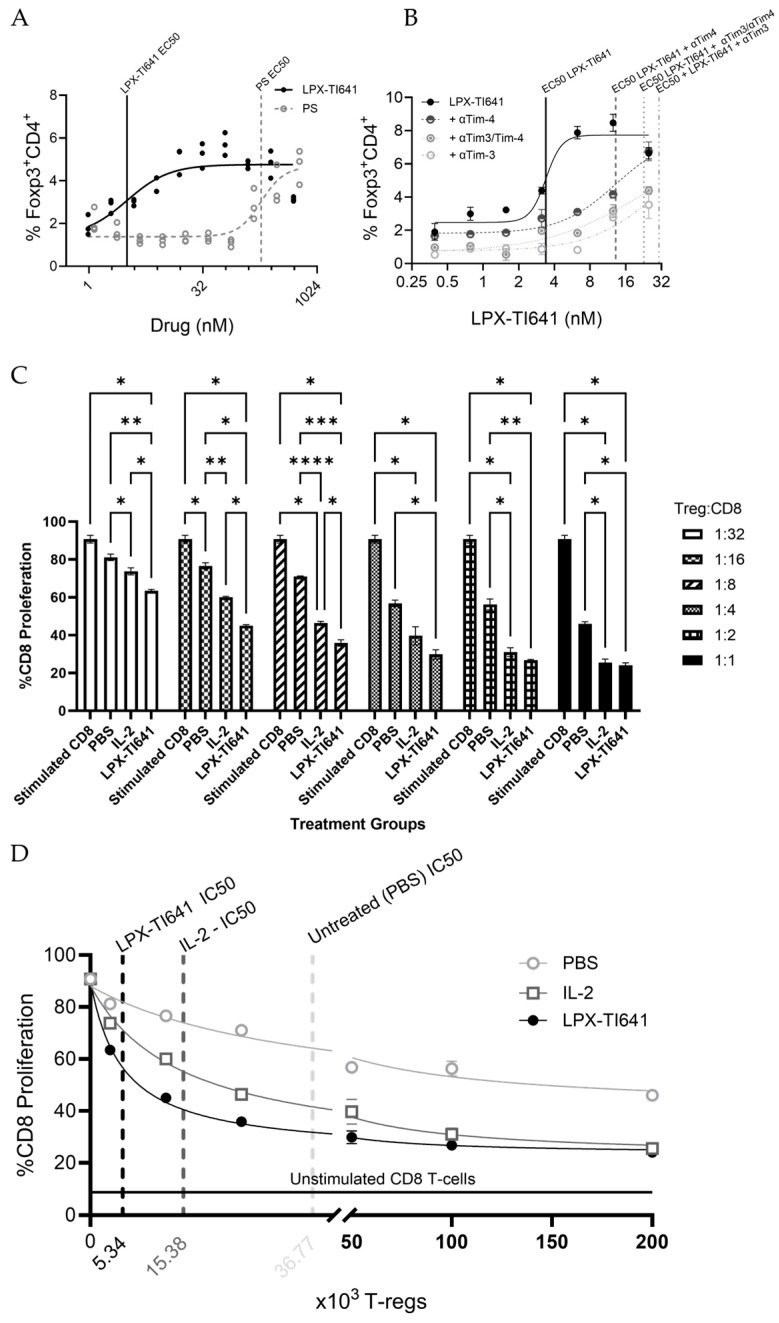
LPX-TI641 Enhances T-reg Frequency and Function in vitro and in vivo. (**A**) Dose-dependent induction of CD4^+^FoxP3^+^ T-regs following 5 days of splenocyte culture with LPX-TI641 or PS. Model fitting yielded an EC_50_ of 3.17 nM for LPX-TI641 and 187 nM for PS, indicating that LPX-TI641 is approximately two orders of magnitude more potent than PS. (**B**) Effect of αTim3 and αTim4 antibodies on LPX-TI641-induced T-reg expansion. Pre-treatment of splenocytes with 20 µg/mL antibodies for 30 min prior to LPX-TI641 exposure. After 5 day of culture, the EC_50_ shifted from 3.38 nM (no antibody) to 30.52 nM (αTim3), 13.16 nM (αTim4), and 22.75 nM (combined), suggesting that LPX-TI641 activity depends on Tim3 and Tim4 signaling. (**C**) T-reg suppressive function assessed in vitro using CD45.1^+^ T-regs isolated from mice treated with LPX-TI641 (10 µg SC, single dose) or IL-2 (500 ng/day IP for 5 days). LPX-TI641-derived T-regs showed significantly greater suppression of CD8^+^ T cell proliferation at all T-reg ratios compared to IL-2- or PBS-derived T-regs. (**D**) IC_50_ values from CD8^+^ T cell proliferation assays co-cultured with T-regs isolated from LPX-TI641-, IL-2-, or PBS-treated mice were 5.33 × 10^3^, 15.38 × 10^3^ and 36.77 × 10^3^ respectively. A *p*-value less than 0.05, 0.01, 0.001, or 0.0001 is shown as *, **, ***, or ****, respectively.

Both LPX-TI641 and PS produced a dose-dependent increase in CD4^+^FoxP3^+^ T cells. However, LPX-TI641 demonstrated significantly greater potency, with an EC_50_ value of 3.17 nM compared to 187 nM for PS—indicating approximately 60-fold higher activity, which is in line with the in silico data suggesting LPX-TI641 has tighter binding to Tim. Figure 4A and Supplementary Figure S1A shows the model fit of the data using a four-parameter dose–response curve.

#### 3.2.2. LPX-TI641 Pharmacology Is Mediated by Tim-3 and Tim-4

Preincubation of splenocytes from naïve C57BL/6 Foxp3^GFP^ mice with α–Tim-3, α–Tim-4, shifted the LPX-TI641 dose–response curve rightward. The EC_50_ increased from 3.38 nM (no antibody) to 30.52 nM (α–Tim-3), 13.16 nM (α–Tim-4), and 22.75 nM (combined), indicating that LPX-TI641′s Treg-expanding activity depends on these receptors (Figure 4B and Supplementary Figure S1B).

#### 3.2.3. T-Regs Suppressive Assay

LPX-TI641–derived Tregs inhibited CD8+ T-cell proliferation significantly at all tested Treg:CD8 ratios, whereas IL-2–derived Tregs showed significant suppression only at 1:8, 1:2, and 1:1 ratio. Tregs from PBS-treated mice had no significant effect. When directly compared, LPX-TI641–derived Tregs produced significantly greater inhibition of percent CD8 proliferation than IL-2–derived Tregs at 1:32, 1:16, and 1:8 ratios; at higher ratios, IL-2 Tregs matched LPX-TI641′s efficacy (Figure 4C).

Fitting CD8 proliferation data yielded IC50 values of 5.33 × 10^3^ (95% CI: 4.56–6.26 × 10^3^) Tregs for LPX-TI641 versus 15.38 × 10^3^ (11.61–20.44 × 10^3^) Tregs for IL-2 (mean, 95% CI). Both LPX-TI641 and IL-2–derived Tregs were more potent suppressors than PBS Tregs. The maximal suppression achieved by LPX-TI641 and IL-2–derived Tregs was approximately 74%, compared to 54% for PBS-derived Tregs (Figure 4D).

### 3.3. Pharmacokinetics and In Vivo Dose–Response Studies

#### 3.3.1. Pharmacokinetics Studies of LPX-TI641

**Figure 5:**
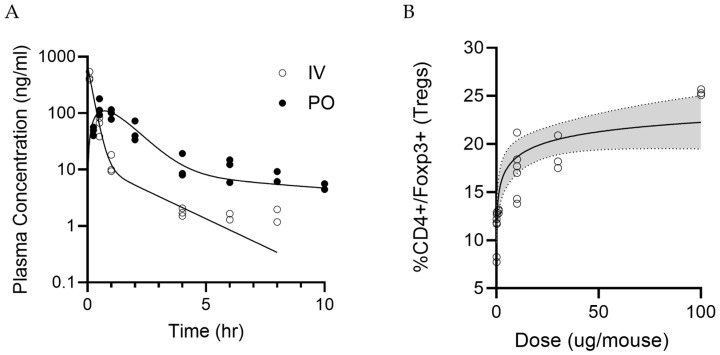
Pharmacokinetics and Dose-Dependent T-reg Induction by LPX-TI641 in Mice. (**A**) Plasma concentration–time profile of LPX-TI641 following intravenous and oral administration in CD1 mice. Oral bioavailability was calculated at 44%. (**B**) Dose-dependent increase in CD4^+^FoxP3^+^ T-regs five days after subcutaneous administration of LPX-TI641 in C57BL/6 mice.

The pharmacokinetic concentration vs. time profile for the IV and oral doses are presented in Figure 5A. Based on NCA, the bioavailability after oral administration was calculated at ~44%.

#### 3.3.2. In Vivo Dose–Response Data of LPX-TI641

The percentage of splenic CD4+/Foxp3+ T-cells increased in a dose-dependent manner from a mean value of 10.7% (SD = 2.2) in the low dose group to 25.4% (SD = 0.31) in the high dose group. The ED50 was estimated at 4.88 μg and ED90 was estimated at 82.1 μg (Figure 5B and Supplementary Figure S2).

### 3.4. LPX-TI641 Controls Disease Progression in PLP and High-Dose MOG-Induced EAE

#### 3.4.1. High-Dose MOG-Induced EAE Model

**Figure 6:**
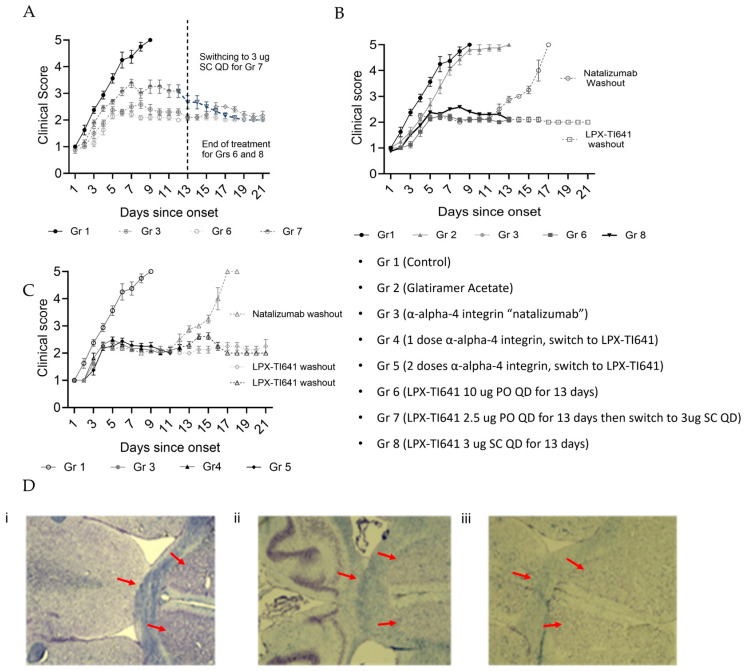
LPX-TI641 Ameliorates Disease in the MOG_35–55_ induced EAE mouse model. (**A**) Clinical scores of mice immunized with MOG_35–55_ and treated at disease onset with varying doses of LPX-TI641 (*n* = 10/group). (**B**) Comparison of clinical EAE scores in mice treated with GA (glatiramer acetate), natalizumab, or LPX-TI641 (*n* = 10/group). (**C**) Clinical scores of EAE mice initially treated with natalizumab and subsequently switched to LPX-TI641 (*n* = 10/group). (**D**) Microscopic imaging (10×) of Luxol Fast Blue staining of brain tissue from EAE mice treated with: (**i**) LPX-TI641 (Gr 6); (**ii**) a single dose of natalizumab (Gr 4); or (**iii**) two doses of natalizumab followed by LPX-TI641 (Gr 5). The LFB images are provided as qualitative illustrations of regions of demyelination and preserved myelin. Arrows highlight those regions for clarity.

In high-dose MOG_35–55_-induced EAE, disease onset occurred on average by day 9 post-immunization. Mice were randomized into treatment groups upon reaching a disease score of 1, with all group assignments completed by day 11 (Figure 1). In the untreated group, animals rapidly progressed, peaking in disease severity by day 9 post-randomization. The severity of disease observed in this high dose MOG_33–55_ is in line with previously reported data using high dose MOG_33–55_ [18]. LPX-TI641 treatment resulted in dose-dependent disease control. Mice receiving 10 µg orally once daily (PO QD) or 3 µg subcutaneously (SC) twice weekly exhibited significantly improved outcomes compared to the 2.5 µg PO QD group, with clinical scores in the higher-dose groups not exceeding 2.5 (Figure 6A).

Glatiramer acetate (GA) failed to control disease after onset, with all mice reaching the study endpoint by day 11, which is in line with previous observations with GA [19]. LPX-TI641 (3 µg SC or 10 µg PO) and natalizumab performed comparably during treatment. However, upon treatment cessation, natalizumab-treated animals experienced disease rebound, while LPX-TI641 maintained disease suppression for at least 13 days post-final dose (Figure 6B). Switching from natalizumab to LPX-TI641 after one or two doses prevented post-washout disease progression, with disease control sustained for 14 days following the final LPX-TI641 dose (Figure 6C).

At the end of the study (day 21 post-onset), brain tissue from EAE mice was examined using Luxol Fast Blue (LFB) staining, a method that highlights intact myelin in blue. This staining helps visualize the extent of myelin preservation or loss; stronger blue staining indicates better-preserved myelin, whereas reduced blue staining suggests myelin damage or demyelination.

Brain sections were analyzed from three groups: mice treated exclusively with LPX-TI641 from disease onset, mice switched to LPX-TI641 after one dose of natalizumab, and mice switched after two doses. As shown in Figure 6D (panels i–iii), LFB staining revealed superior myelin preservation in the group treated with LPX-TI641 from the beginning (Figure 6D panel i), compared to those that began treatment with natalizumab and switched later (Figure 6D panels ii and iii). Among the combination-treatment groups, myelin integrity was better preserved when the switch to LPX-TI641 occurred after a single dose rather than two doses of natalizumab.

#### 3.4.2. PLP-Induced EAE Model

In the PLP-induced EAE model, disease onset began around day 9, and treatment commenced for all groups on day 15. The untreated group peaked in disease severity by day 19 (mean score: 3.5), compared to 3.0 and 2.8 in natalizumab- and LPX-TI641-treated groups, respectively. All groups entered remission by day 25, with lower mean clinical scores in treated groups (score of 1.0) versus untreated (score of 1.5).

**Figure 7:**
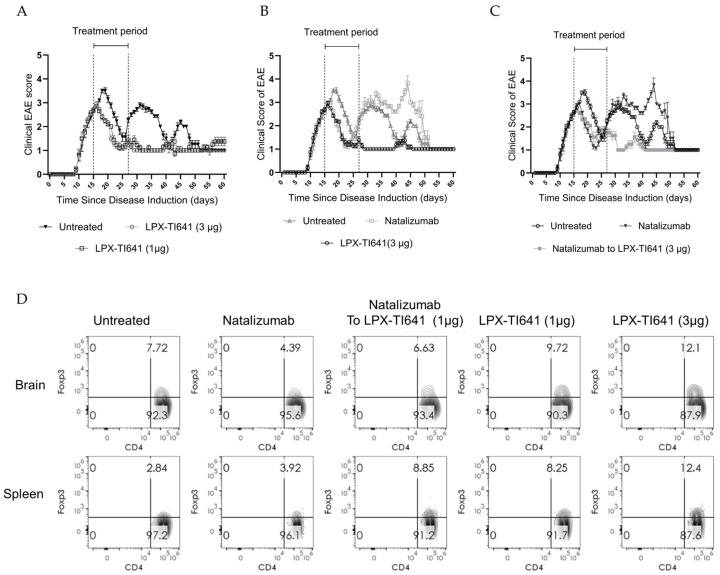
LPX-TI641 Suppresses Relapse in the PLP-Induced EAE Mouse Model. (**A**) Clinical scores of mice immunized with PLP and treated on day 15 post-immunization with varying doses of LPX-TI641 (*n* = 10/group). (**B**) Comparison of EAE clinical scores in mice treated with natalizumab or LPX-TI641 (*n* = 10/group). (**C**) Clinical scores of EAE mice initially treated with natalizumab and subsequently switched to LPX-TI641 (*n* = 10/group). (**D**) Flow cytometry plots showing CD4^+^Foxp3^+^ T-reg expression in brain (**top**) and spleen (**bottom**) at day 21 post-immunization across different treatment groups.

Untreated animals experienced relapses on days 31 and 45, followed by remission around day 53 (Figure 7A). Natalizumab-treated mice relapsed post-treatment and eventually mirrored the disease progression of untreated controls (Figure 4B). LPX-TI641 treatment (1 µg or 3 µg SC) resulted in sustained remission from day 16 through study end, with no observed relapses (Figure 7A,B). Notably, administering LPX-TI641 between days 18–26, following a single dose of natalizumab, prevented severe relapse and achieved better disease control compared to natalizumab alone (Figure 7C).

T-cell profiling of brain tissue showed a dose-dependent increase in FoxP3^+^ Tregs in LPX-TI641-treated animals compared to controls, whereas natalizumab-treated animals had the lowest brain Treg levels (Figure 7D, top). Similar trends were observed in the spleen, with LPX-TI641 inducing the highest Treg levels and natalizumab showing the least effect (Figure 7D, bottom).

#### 3.4.3. LPX-TI641 Is Effective in an Escalation Treatment Paradigm

**Figure 8:**
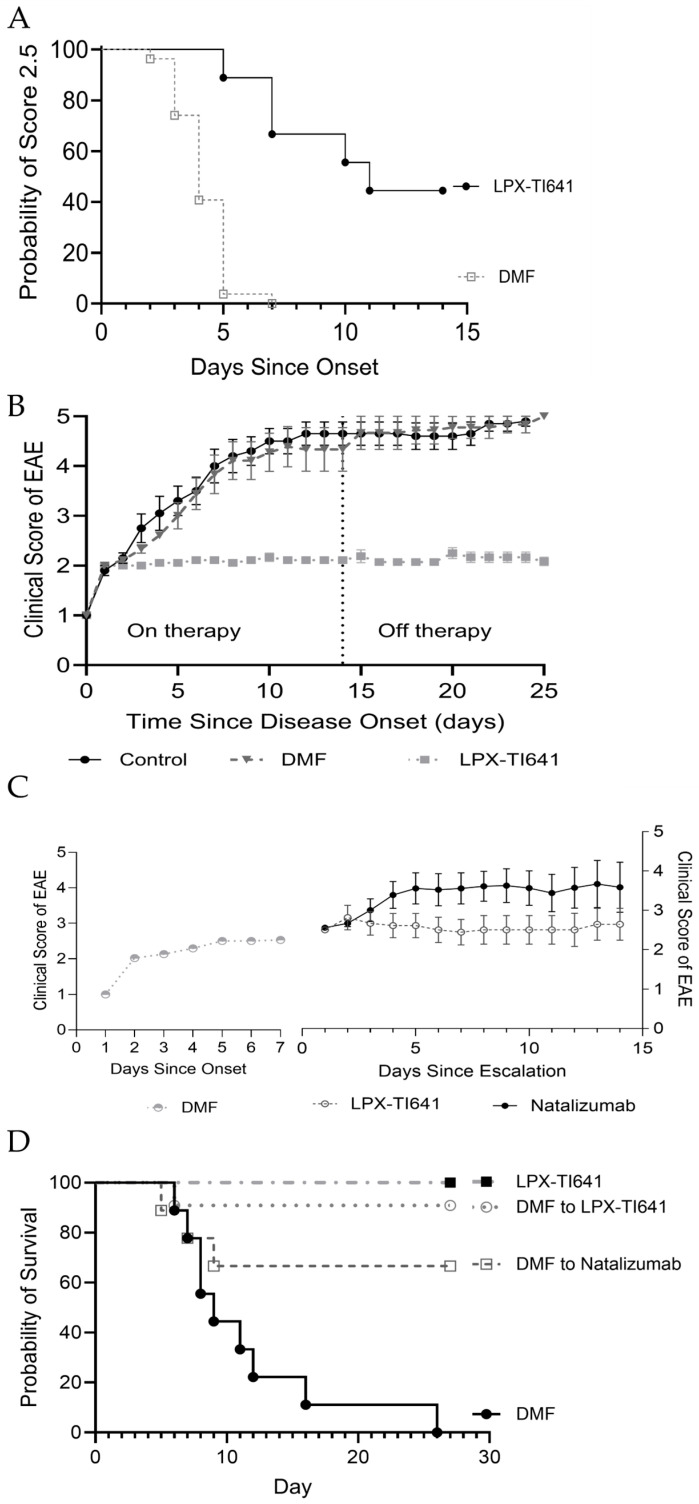
LPX-TI641 in an Escalation Treatment Paradigm in the MOG-Induced EAE Mouse Model. (**A**). Kaplan–Meier plot showing the efficacy of LPX-TI641 in delaying or preventing disease progression compared to dimethyl fumarate (DMF). (**B**) EAE clinical scores in mice treated with DMF vs. LPX-TI641 (*n* = 10/group). (**C**) Clinical scores for mice treated with DMF followed by escalation to either LPX-TI641 or natalizumab (*n* = 10/group). (**D**) Survival curves comparing treatment groups following therapeutic escalation paradigm.

In a treatment escalation model using MOG_35–55_-induced EAE, animals that reached a disease score of 1 were randomized to dimethyl fumarate (DMF, *n* = 30), untreated (*n* = 10), or LPX-TI641 (*n* = 9) (Figure 2). Within 7 days, all DMF-treated mice progressed to a score of 2.5. In contrast, only 5 of 9 LPX-TI641-treated animals reached this score (*p* = 0.001, Fisher’s exact test) (Figure 8A,B).

Upon reaching a score of 2.5, animals in the DMF cohort were re-randomized to continue DMF (*n* = 9), or switch to natalizumab (*n* = 9) or LPX-TI641 (*n* = 9). Animals continuing DMF progressed similarly to untreated controls, all reaching a score of 5 by study end. Switching to LPX-TI641 significantly improved outcomes, with a mean clinical score of 2.6 (SD = 1.1), compared to 3.6 (SD = 1.6) in the natalizumab-switch group (Figure 8C). Animals started on LPX-TI641 from onset had the best disease control both during and after therapy.

Survival analysis showed 100% survival in the LPX-TI641 group vs. 0% in the continued DMF group by day 27. Among switch groups, 8/9 mice switching to LPX-TI641 survived, compared to 6/9 in the natalizumab-switch group (Figure 8D).

#### 3.4.4. LPX-TI641 Outperforms Natalizumab in an Induction/Maintenance Paradigm

**Figure 9:**
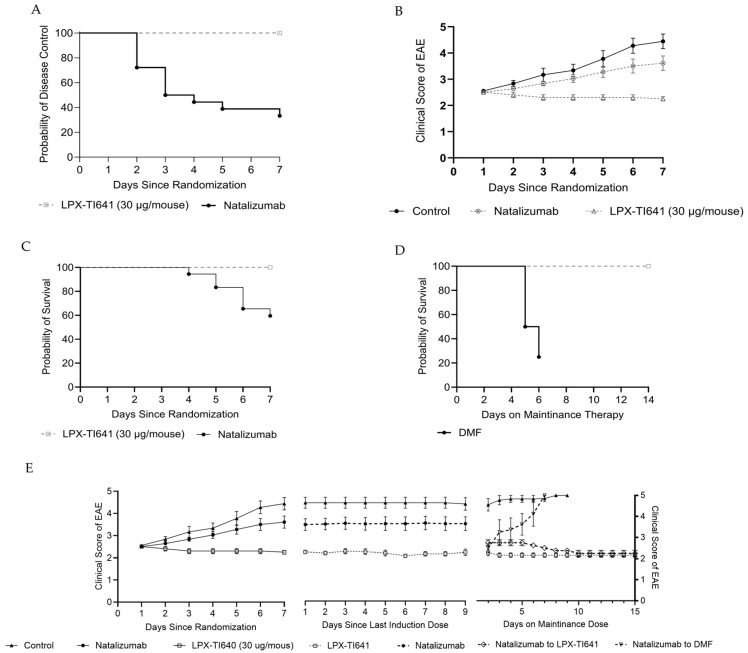
LPX-TI641 in an Induction/Maintenance Paradigm in the MOG-Induced EAE Mouse Model. (**A**) Kaplan–Meier plot showing the efficacy of LPX-TI641 in controlling disease compared to natalizumab. (**B**) EAE clinical scores in mice treated with natalizumab or LPX-TI641 (*n* = 10/group). (**C**) Survival curves for mice randomized to receive LPX-TI641 or natalizumab during disease progression. (**D**) Survival curves during maintenance therapy with LPX-TI641 or natalizumab. (**E**) Clinical scores of EAE in mice initially treated with natalizumab and then transitioned to maintenance therapy with either dimethyl fumarate (DMF) or LPX-TI641.

In a second model mimicking induction/maintenance treatment, animals reaching a score of 2.5 were randomized to natalizumab (*n* = 18), LPX-TI641 (*n* = 9), or untreated (*n* = 9) groups (Figure 3). Of the natalizumab-treated animals, 12 progressed beyond score 2.5 and only 11 survived induction period. In contrast, none of the LPX-TI641-treated animals exceeded this threshold (Figure 9A), and all survived (Figure 9C).

Post-induction period, surviving natalizumab-treated animals were re-randomized to maintenance with either DMF (*n* = 4) or LPX-TI641 (*n* = 4). Only 1/4 animals survived in the DMF group vs. 4/4 in the LPX-TI641 group (Figure 9D). On day 7 of maintenance, the clinical score was significantly lower in the LPX-TI641 group (2.5 ± 0) compared to DMF (4.37 ± 1.2) (Figure 9E).

Animals treated continuously with LPX-TI641 (induction + maintenance) maintained stable clinical scores throughout the 14-day maintenance period, ending with a mean score of 2.2 (SD = 0.3), demonstrating consistent and durable disease control.

### 3.5. LPX-TI641 Pharmacology in MS-Patient (PwMS) Derived PBMC’s

#### 3.5.1. Demographics and Current Treatments of PwMS Enrolled in the Study

Whole blood samples were collected from six female patients with a confirmed diagnosis of multiple sclerosis (MS), ranging in age from 44 to 60 years. The majority of participants had a body mass index (BMI) greater than 30. Among these patients, three were diagnosed with relapsing-remitting MS (RRMS), one with active secondary progressive MS (SPMS), and the MS subtype for two patients was undocumented. All participants were undergoing disease-modifying therapy (DMT), which included interferon beta-1a, natalizumab, dimethyl fumarate, and glatiramer acetate.

#### 3.5.2. Reduced Baseline Tim3^+^ Regulatory T Cells in PwMS Compared to Healthy Controls

**Figure 10:**
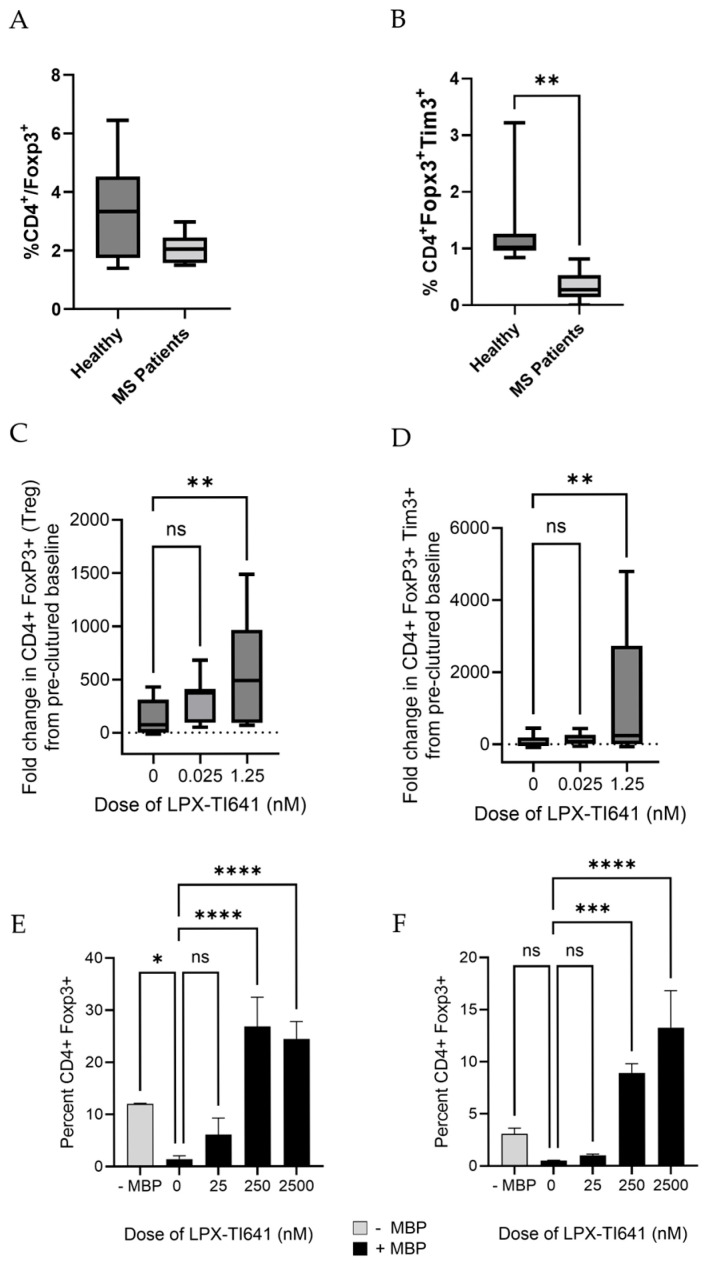
LPX-TI641 Enhances Regulatory T Cell Responses in Patients with Multiple Sclerosis (PwMS) after 5 days of culture. (**A**) Comparison of total CD4^+^FoxP3^+^ T-regs between healthy donors (*n* = 9) and patients with MS (PwMS, *n* = 6). (**B**) Comparison of total Tim-3^+^ T-regs between healthy donors (*n* = 9) and patients with MS (PwMS, *n* = 6). (**C**) Dose-dependent expansion of total T-regs in PBMCs from PwMS after 5 days of culture with ascending doses of LPX-TI641. (**D**) Dose-dependent expansion of total Tim-3^+^ T-regs in PBMCs from PwMS after 5 days of culture with ascending doses of LPX-TI641. (**E**,**F**) T-reg expression in PBMCs from Subjects 1 and 4, respectively without the addition of myelin basic protein (−MBP) and with the MBP with and without increasing concentration of LPX-TI641. A *p*-value less than 0.05, 0.01, 0.001, or 0.0001 is shown as *, **, ***, or ****, respectively.

At baseline, the mean percentage of CD4^+^FoxP3^+^ regulatory T cells (Tregs) in peripheral blood mononuclear cells (PBMCs) were 3.4% (SD = 1.8) in healthy controls (HCs, *n* = 7) and 2.1% (SD = 0.54) in people with MS (PwMS, *n* = 6). Although this difference was not statistically significant, it is consistent with previously reported findings (35) (Figure 10A). Notably, the frequency of CD4^+^FoxP3^+^Tim3^+^ Tregs (Tim3^+^ Tregs) was significantly lower in PwMS compared to HCs, with mean percentages of 0.3% (SD = 0.3) and 1.3% (SD = 0.8), respectively (Figure 10B).

#### 3.5.3. LPX-TI641 Enhances Treg and Tim3^+^ Treg Populations in PwMS-Derived PBMCs

Ex vivo treatment of PBMCs derived from PwMS with LPX-TI641 led to a dose-dependent increase in both total FoxP3^+^ Tregs and the Tim3^+^ Treg subset compared to untreated control cultures. At the highest tested concentration, LPX-TI641 increased CD4^+^/FoxP3^+^ Tregs and CD4^+^/FoxP3^+^/Tim3^+^ Tregs by approximately 6-fold and 2-fold from pre-cultured baseline, respectively (Figure 10C,D).

#### 3.5.4. LPX-TI641 Reverses MBP-Induced Suppression of Tregs in Autoreactive Patients

In a recall response assay using the myelin basic protein (MBP) peptide MBP_87–99_, 2 out of 6 subjects (subject 1 and subject 4) exhibited a 50% or greater reduction in CD4^+^FoxP3^+^ Tregs, suggesting the presence of MBP-autoreactive T cells within their repertoire (Figure 10E for subject 1 and 10F for subject 4). Treatment of PBMCs from these two MBP-responsive subjects with LPX-TI641 resulted in a dose-dependent reversal of MBP_87–99_-mediated suppression of both total Tregs and the Tim3^+^ Treg subset (Figure 10E,F), supporting the potential of LPX-TI641 to restore immune tolerance in MS patients with autoreactive T-cell populations.

## 4. Discussion

Immune tolerance induction remains promising for the treatment of autoimmune conditions but faces challenges in practicality and scalability, especially for diseases with multiple antigens such as MS or those with undefined antigens. Most existing tolerance induction strategies rely on a priori identification of disease-relevant antigens or epitopes [9] and subsequent administration of antigenic material in a tolerogenic context requiring complex nanoparticles or ex vivo manipulation of cells [7,20,21].

In this study, we introduce LPX-TI641, an orally bioavailable, clinical stage, small-molecule agonist of Tim-3 and Tim-4 receptors that promotes immune tolerance by expanding Foxp3^+^ regulatory T cells (Tregs), including the stable and highly suppressive Foxp3^+^Tim3^+^ subset. LPX-TI641 mimics phosphatidylserine (PS), a natural Tim-receptor ligand involved in apoptotic cell clearance and self-tolerance [22], but exhibits markedly greater potency. In splenocyte assays, LPX-TI641 was 60-fold more potent than PS at inducing CD4^+^Foxp3^+^ Tregs; this effect was abrogated by Tim-3 or Tim-4 blockade, confirming receptor specificity. Moreover, Tregs from LPX-TI641-treated mice suppressed CD8^+^ T-cell proliferation significantly at all tested Treg:CD8 ratios, whereas IL-2-derived Tregs were effective only at higher ratios. LPX-TI641–derived Tregs had a lower IC_50_ compared to IL-2–derived or PBS-derived Tregs. These results demonstrate the superior capacity of LPX-TI641 to induce highly suppressive Tregs and support its potential as a therapeutic agent for promoting immune tolerance.

The therapeutic efficacy of LPX-TI641 was tested in two experimental autoimmune encephalomyelitis (EAE) models that mimic features of MS. In the MOG_35–55_-induced EAE model, LPX-TI641 demonstrated robust symptom control in a dose-dependent manner. Animals treated with higher doses (10 µg oral QD or 3 µg SC twice weekly) showed better outcomes than those on the low-dose oral regimen. Notably, when animals were switched from a low-dose regimen to a higher dose during disease progression, disease scores improved, which may suggest either enhanced inflammation control or support for tissue repair by Tregs, which have been implicated in myelin restoration [23]. When compared to glatiramer acetate (GA), a low-efficacy DMT, LPX-TI641 provided superior disease control post-onset, consistent with GA’s known limitations in therapeutic EAE settings [19]. Importantly, LPX-TI641 matched the efficacy of natalizumab during active treatment but sustained symptom control even after drug washout—something not observed with natalizumab. Additionally, switching from natalizumab to LPX-TI641 after one or two doses preserved disease remission and improved myelin integrity histologically, with earlier switches correlating with better myelin preservation.

In the PLP_139–151_-induced EAE model, which recapitulates epitope spreading, a hallmark of MS progression, LPX-TI641 treatment initiated during the acute phase (day 15–26) effectively prevented both the first and second relapses. In contrast, natalizumab-treated animals experienced relapses after discontinuation, with more severe secondary relapses than untreated controls. LPX-TI641 not only prevented relapses but also maintained disease remission beyond the treatment window, despite the drug’s short half-life in mice. This suggests a durable pharmacodynamic effect, likely mediated by the sustained presence of Tregs in circulation. Given that treatment ceased before the onset of the first relapse, these data support a mechanism of immune tolerance, rather than simple immunosuppression or T-cell trafficking blockade.

We also explored therapeutic positioning of LPX-TI641 using treatment escalation and induction/maintenance paradigms. In the treatment escalation model, mice were initially treated with dimethyl fumarate (DMF), a moderate-efficacy DMT. Animals that failed DMF treatment were switched to either natalizumab or LPX-TI641. While both agents improved disease control, LPX-TI641 demonstrated higher survival and better clinical scores post-switch. The most effective group in the study consisted of animals started on LPX-TI641 from disease onset, further underscoring the benefit of early intervention.

In the induction/maintenance paradigm, animals were treated at a late disease stage (clinical score of 2.5). LPX-TI641 was superior to natalizumab in both disease control and survival during induction. Furthermore, animals switched to LPX-TI641 for maintenance therapy showed continued clinical improvement, while those switched to DMF for maintenance therapy relapsed and experienced high mortality. These findings suggest that LPX-TI641 is not only effective as an induction agent but may also support long-term immune homeostasis when used for maintenance.

Importantly, the immune tolerance–inducing potential of LPX-TI641 was validated ex vivo using PBMCs derived from PwMS. Compared to healthy controls, PwMS had lower baseline levels of Foxp3^+^Tim3^+^ Tregs. LPX-TI641 treatment of patient-derived PBMCs significantly expanded both total Foxp3^+^ Tregs and the Foxp3^+^Tim3^+^ subset in a dose-dependent manner. This is notable because the Tim3^+^ Treg population is believed to play a key role in durable immune regulation and is impaired in MS [11,24,25,26]. In a recall response study using the myelin basic protein (MBP_87–99_) peptide, LPX-TI641 reversed antigen-induced suppression of Tregs in responsive patient samples, further supporting its ability to restore tolerance even in the context of autoreactive T-cell memory.

Collectively, our data demonstrates that LPX-TI641, a Tim-3/Tim-4 receptor agonist, promotes immune tolerance and restores immune balance by expanding regulatory T-cell populations. It exhibited robust efficacy across multiple therapeutic EAE models in an antigen-independent manner. Importantly, effects of LPX-TI641persisted after treatment cessation and effectively expanded Tim-3^+^ Tregs derived from patient immune cells. Unlike tolerance strategies that require rapamycin or DMT’s such as dexamethasone, Tim agonists does not broadly suppress TLR signaling pathways [14]. Taken together, these findings support that Tim-agonism, and LPX-TI641 in particular, holds promise as a non-immune suppressant, durable, and practical immunomodulatory therapy for autoimmune diseases such as multiple sclerosis. LPX-TI641 is currently being evaluated clinically in a series of phase I and phase Ib studies to evaluate its safety, tolerability and pharmacokinetics.

## Figures and Tables

**Table 1:** In silico generated binding affinity and dissociation constants for Tim natural ligands and novel LPX molecules.

	Tested Compound		
Reported Parameter	Phosphatidylserine	LPX3	LPX-TI641
∆G (kcal/mol)	−6.0 to −5.1	−5.2 to −4.5	−8.2 to −6.2
Kd (M)	3.95 × 10^−5^ to 1.81 × 10^−4^	1.53 × 10^−4^ to 4.99 × 10^−4^	9.61 × 10^−7^ to 2.82 × 10^−5^

## Data Availability

The data presented in this study is available on request from the corresponding author. (The data are not publicly available due to potential IP and confidentiality restrictions).

## Supplementary Materials

The following supporting information can be downloaded at: https://www.mdpi.com/article/10.3390/pharmaceutics17111402/s1, Figure S1: Flow Cytometry (FACS) Raw Data of In Vitro–Cultured Splenocytes. Figure S2: Gating Strategy and Raw FACS Data of Splenocytes from In Vivo LPX-TI641–Treated Mice.

## Abbreviations

The following abbreviations are used in this manuscript:

CFA	Complete Freund’s Adjuvant
CNS	Central nervous system
DMF	Dimethyl fumarate
DMT	Disease-modifying therapy
EAE	Experimental autoimmune encephalomyelitis
EC50	Half-maximal effective concentration (EC50)
Foxp3^+^	Forkhead box P3, a specific transcription factor for Treg (a signature of regulatory T cells)
GA	Glatiramer Acetate
MBP	Myelin basic protein
MOG_35–55_	Myelin oligodendrocyte glycoprotein (35–55), a 20-amino acid peptide of MOG protein a component of CNS myelin sheath.
MS	Multiple Sclerosis
PBMC	Peripheral blood mononuclear cell
PD	Pharmacodynamics
PK	Pharmacokinetics
PLP_139–151_	A synthetic myelin proteolipid protein fragment, known to induce relapsing remitting EAE
QD	Once a day dosing
SC	Subcutaneous dosing
TCR	T-cell receptor
Tim3	T cell/transmembrane, immunoglobulin, and mucin 3
Treg	Regulatory T-cells, known to regulate immune response and suppress pathogenic T-cell
