# Supplementary material for: LPX-TI641, a Tim3/4 Agonist, Induces Long-Term Immune Tolerance in Multiple Sclerosis Models
Source: Pharmaceutics. 2025 Oct 30;17(11):1402. doi: 10.3390/pharmaceutics17111402 (PMC12655510; doi:10.3390/pharmaceutics17111402)

Supplementary data

Supplementary Figure S1: Raw data of FACS plots of in vitro culture splecnocytes

A

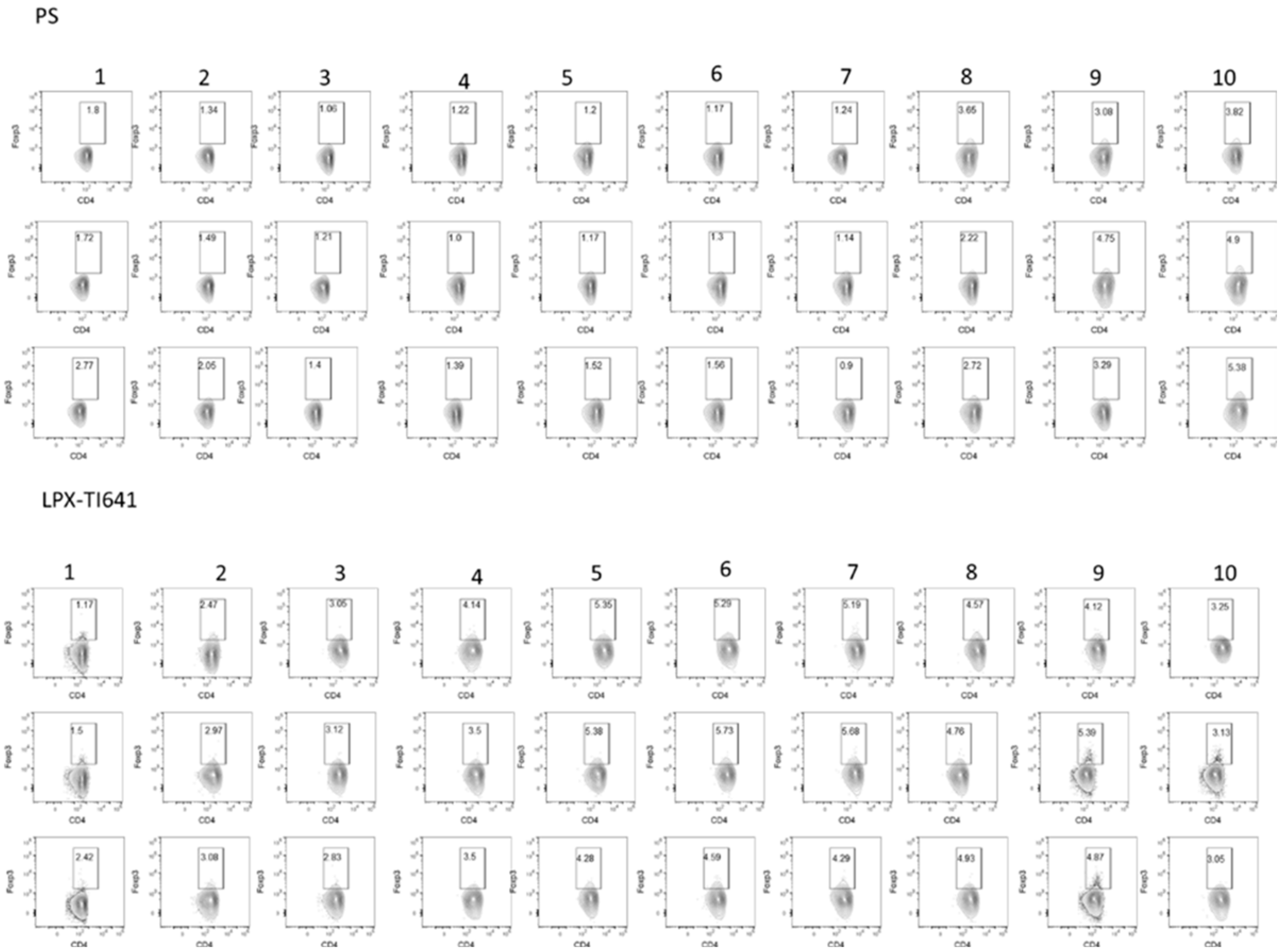

B

LPX-TI641 (ng/ml) + anti-Tim3 (20ug/ml)

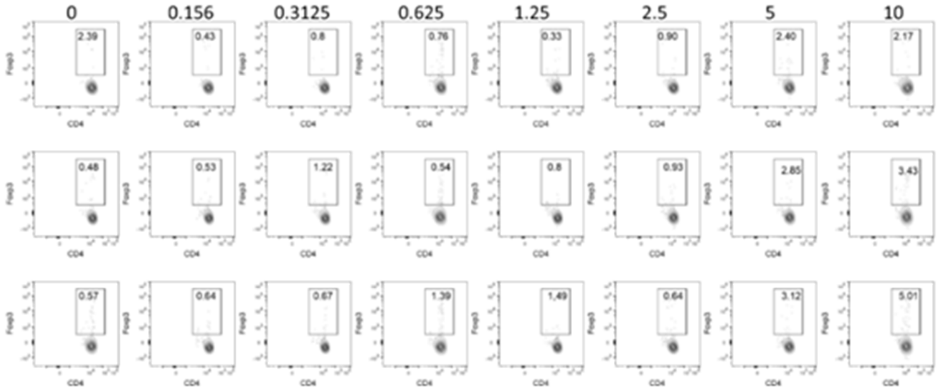

LPX-TI641 (ng/ml) + anti-Tim4 (20ug/ml)

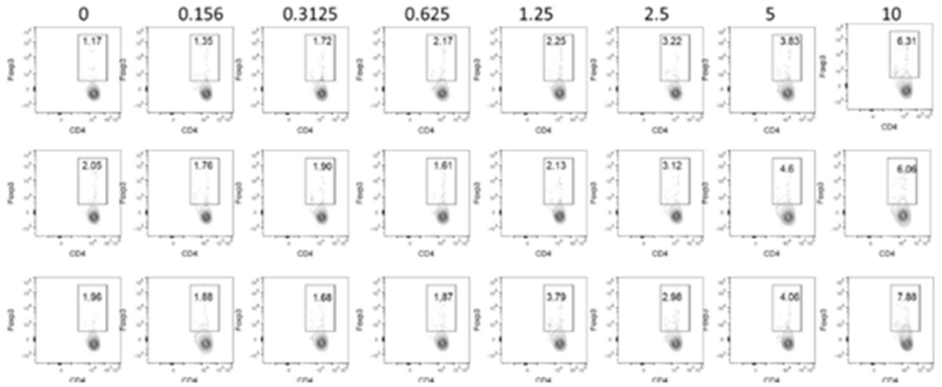

LPX-TI641 (ng/ml) + anti-Tim3 (20ug/ml) + anti-Tim4 (20ug/ml)

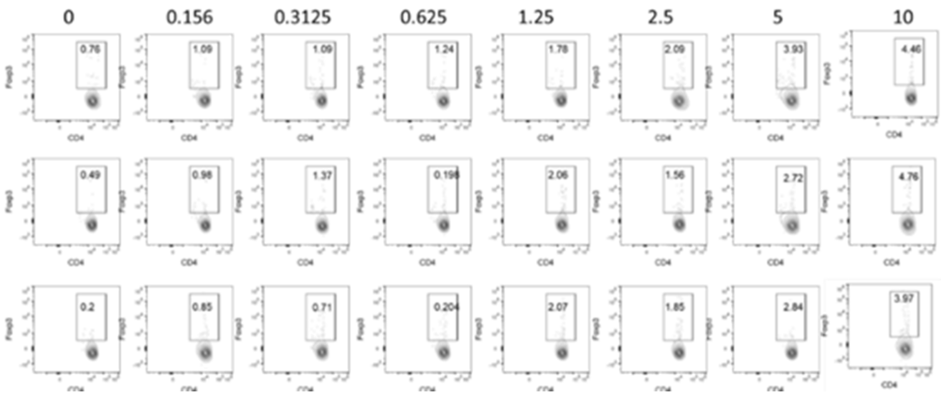

Supplementary Figure S2. Gating strategy and raw data of FACS plots of splenocytes from in vivo-treated mice with LPX-TI641

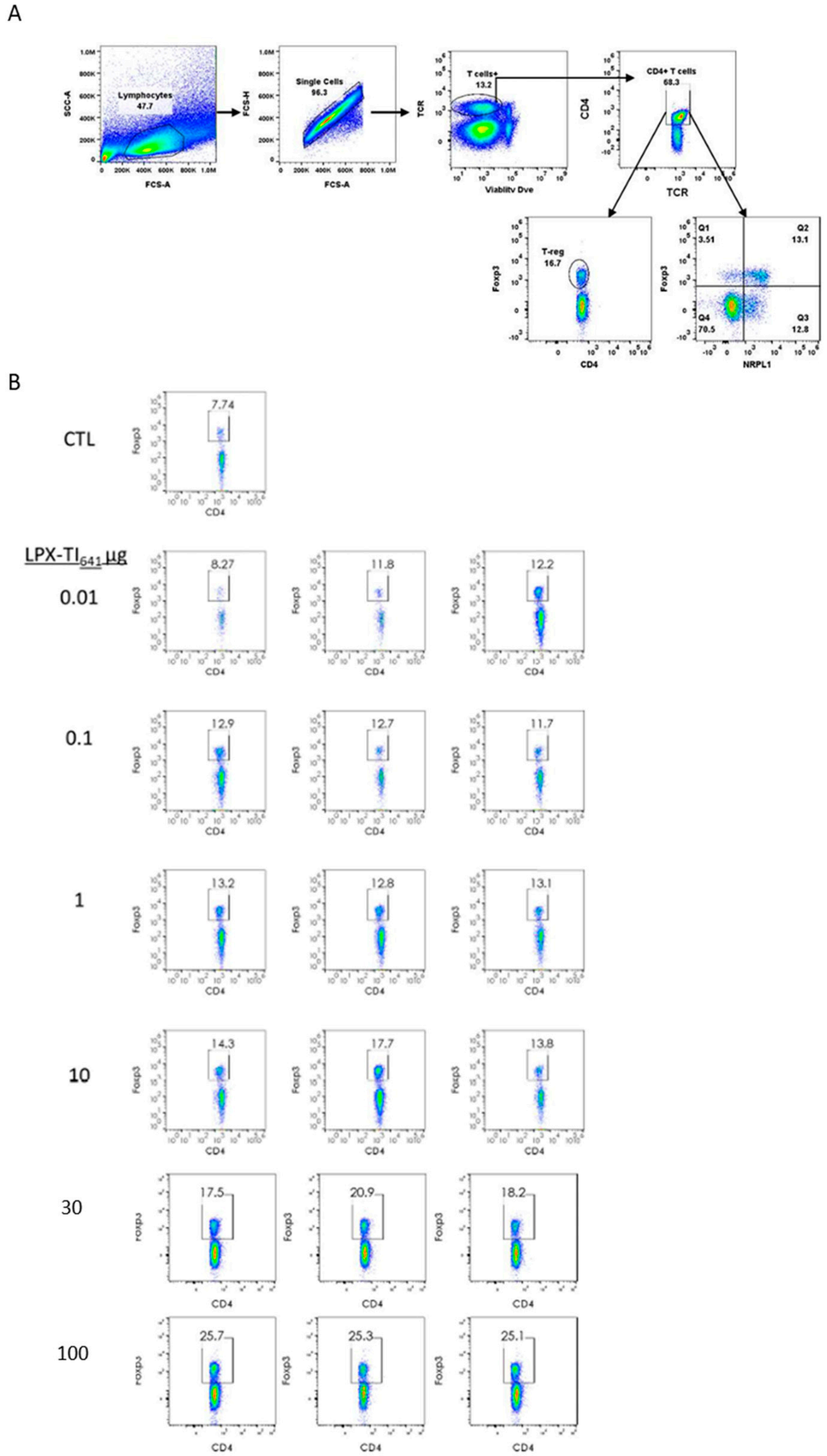

Supplement: Supplementary file 1 [file pharmaceutics-17-01402-s001.zip › pharmaceutics-3934482-supplementary.pdf]
